# Supplementary material for: Modeling glioblastoma heterogeneity as a dynamic network of cell states
Source: Mol Syst Biol. 2021 Sep 16;17(9):e10105. doi: 10.15252/msb.202010105 (PMC8444284; doi:10.15252/msb.202010105)
Supplement: Supplementary file 6 — Source Data for Figure 5 [file MSB-17-e10105-s004.zip › Figure5A_sourcedata/GSEA_3071/hallmarks_stateVI.GseaPreranked.1623742557031/HALLMARK_EPITHELIAL_MESENCHYMAL_TRANSITION.html]

Details for gene set HALLMARK\_EPITHELIAL\_MESENCHYMAL\_TRANSITION[GSEA]

|  || Dataset | state63071 |
| Phenotype | NoPhenotypeAvailable |
| Upregulated in class | na\_pos |
| GeneSet | HALLMARK\_EPITHELIAL\_MESENCHYMAL\_TRANSITION |
| Enrichment Score (ES) | 0.27260318 |
| Normalized Enrichment Score (NES) | 1.9023639 |
| Nominal p-value | 0.0 |
| FDR q-value | 0.013854754 |
| FWER p-Value | 0.047 |
Table: GSEA Results Summary

  

Fig 1: Enrichment plot: HALLMARK\_EPITHELIAL\_MESENCHYMAL\_TRANSITION      
 Profile of the Running ES Score & Positions of GeneSet Members on the Rank Ordered List

  

| PROBE | GENE SYMBOL | GENE\_TITLE | RANK IN GENE LIST | RANK METRIC SCORE | RUNNING ES | CORE ENRICHMENT || 1 | SERPINE1 |  |  | 0 | 1.673 | 0.0253 | Yes |
| 2 | TGM2 |  |  | 3 | 1.367 | 0.0455 | Yes |
| 3 | DKK1 |  |  | 9 | 1.134 | 0.0612 | Yes |
| 4 | THBS1 |  |  | 12 | 1.097 | 0.0773 | Yes |
| 5 | VEGFC |  |  | 19 | 1.035 | 0.0913 | Yes |
| 6 | MFAP5 |  |  | 22 | 1.009 | 0.1060 | Yes |
| 7 | LOX |  |  | 29 | 0.968 | 0.1190 | Yes |
| 8 | PLAUR |  |  | 34 | 0.936 | 0.1320 | Yes |
| 9 | FSTL3 |  |  | 41 | 0.912 | 0.1442 | Yes |
| 10 | IL32 |  |  | 46 | 0.880 | 0.1564 | Yes |
| 11 | PTX3 |  |  | 56 | 0.860 | 0.1668 | Yes |
| 12 | BDNF |  |  | 65 | 0.828 | 0.1771 | Yes |
| 13 | ITGA2 |  |  | 71 | 0.810 | 0.1880 | Yes |
| 14 | DAB2 |  |  | 78 | 0.788 | 0.1982 | Yes |
| 15 | INHBA |  |  | 80 | 0.781 | 0.2098 | Yes |
| 16 | GLIPR1 |  |  | 81 | 0.781 | 0.2216 | Yes |
| 17 | GADD45B |  |  | 90 | 0.758 | 0.2308 | Yes |
| 18 | TAGLN |  |  | 100 | 0.737 | 0.2394 | Yes |
| 19 | BASP1 |  |  | 101 | 0.737 | 0.2506 | Yes |
| 20 | QSOX1 |  |  | 187 | 0.609 | 0.2358 | Yes |
| 21 | MYL9 |  |  | 195 | 0.605 | 0.2430 | Yes |
| 22 | GADD45A |  |  | 200 | 0.603 | 0.2510 | Yes |
| 23 | EDIL3 |  |  | 219 | 0.589 | 0.2548 | Yes |
| 24 | GEM |  |  | 233 | 0.577 | 0.2599 | Yes |
| 25 | SNAI2 |  |  | 310 | 0.536 | 0.2466 | Yes |
| 26 | FBN1 |  |  | 311 | 0.536 | 0.2547 | Yes |
| 27 | JUN |  |  | 321 | 0.527 | 0.2601 | Yes |
| 28 | LGALS1 |  |  | 323 | 0.526 | 0.2678 | Yes |
| 29 | COL6A2 |  |  | 335 | 0.521 | 0.2726 | Yes |
| 30 | SFRP1 |  |  | 426 | 0.479 | 0.2544 | No |
| 31 | TNFAIP3 |  |  | 445 | 0.473 | 0.2565 | No |
| 32 | IGFBP4 |  |  | 571 | 0.433 | 0.2278 | No |
| 33 | TPM1 |  |  | 574 | 0.432 | 0.2338 | No |
| 34 | TPM2 |  |  | 595 | 0.427 | 0.2346 | No |
| 35 | TFPI2 |  |  | 727 | 0.391 | 0.2035 | No |
| 36 | NT5E |  |  | 787 | 0.379 | 0.1926 | No |
| 37 | PVR |  |  | 839 | 0.370 | 0.1838 | No |
| 38 | CAPG |  |  | 851 | 0.368 | 0.1862 | No |
| 39 | LOXL1 |  |  | 879 | 0.364 | 0.1841 | No |
| 40 | VIM |  |  | 897 | 0.361 | 0.1848 | No |
| 41 | SDC4 |  |  | 1013 | 0.343 | 0.1575 | No |
| 42 | CDH11 |  |  | 1024 | 0.341 | 0.1598 | No |
| 43 | MATN3 |  |  | 1201 | 0.315 | 0.1149 | No |
| 44 | RGS4 |  |  | 1209 | 0.314 | 0.1177 | No |
| 45 | EMP3 |  |  | 1278 | 0.306 | 0.1031 | No |
| 46 | TIMP3 |  |  | 1290 | 0.305 | 0.1046 | No |
| 47 | MEST |  |  | 1294 | 0.304 | 0.1084 | No |
| 48 | NTM |  |  | 1320 | 0.301 | 0.1059 | No |
| 49 | IL15 |  |  | 1353 | 0.297 | 0.1013 | No |
| 50 | FGF2 |  |  | 1557 | 0.274 | 0.0481 | No |
| 51 | LOXL2 |  |  | 1608 | 0.268 | 0.0381 | No |
| 52 | FN1 |  |  | 1654 | 0.264 | 0.0294 | No |
| 53 | FLNA |  |  | 1737 | 0.257 | 0.0101 | No |
| 54 | TGFBR3 |  |  | 1846 | -0.256 | -0.0165 | No |
| 55 | GPC1 |  |  | 1876 | -0.261 | -0.0208 | No |
| 56 | COL11A1 |  |  | 1919 | -0.267 | -0.0286 | No |
| 57 | SAT1 |  |  | 1952 | -0.271 | -0.0335 | No |
| 58 | GPX7 |  |  | 1999 | -0.278 | -0.0423 | No |
| 59 | VEGFA |  |  | 2104 | -0.298 | -0.0672 | No |
| 60 | SERPINE2 |  |  | 2128 | -0.302 | -0.0691 | No |
| 61 | COL5A2 |  |  | 2160 | -0.307 | -0.0732 | No |
| 62 | APLP1 |  |  | 2259 | -0.325 | -0.0960 | No |
| 63 | THY1 |  |  | 2265 | -0.326 | -0.0925 | No |
| 64 | ECM1 |  |  | 2309 | -0.334 | -0.0995 | No |
| 65 | MMP14 |  |  | 2440 | -0.365 | -0.1307 | No |
| 66 | ITGAV |  |  | 2476 | -0.371 | -0.1350 | No |
| 67 | LAMC1 |  |  | 2519 | -0.382 | -0.1411 | No |
| 68 | MMP2 |  |  | 2550 | -0.388 | -0.1437 | No |
| 69 | BGN |  |  | 2551 | -0.388 | -0.1378 | No |
| 70 | FZD8 |  |  | 2592 | -0.399 | -0.1430 | No |
| 71 | DST |  |  | 2614 | -0.404 | -0.1428 | No |
| 72 | MATN2 |  |  | 2673 | -0.417 | -0.1529 | No |
| 73 | PDLIM4 |  |  | 2678 | -0.419 | -0.1477 | No |
| 74 | TGFBI |  |  | 2679 | -0.419 | -0.1414 | No |
| 75 | LUM |  |  | 2775 | -0.448 | -0.1614 | No |
| 76 | MCM7 |  |  | 2808 | -0.460 | -0.1635 | No |
| 77 | GAS1 |  |  | 2953 | -0.515 | -0.1964 | No |
| 78 | EFEMP2 |  |  | 3010 | -0.546 | -0.2039 | No |
| 79 | ID2 |  |  | 3026 | -0.554 | -0.1998 | No |
| 80 | DCN |  |  | 3027 | -0.554 | -0.1914 | No |
| 81 | FBN2 |  |  | 3088 | -0.579 | -0.1995 | No |
| 82 | SGCB |  |  | 3123 | -0.598 | -0.2001 | No |
| 83 | PFN2 |  |  | 3215 | -0.685 | -0.2154 | No |
| 84 | ELN |  |  | 3269 | -0.728 | -0.2194 | No |
| 85 | PRRX1 |  |  | 3288 | -0.749 | -0.2131 | No |
| 86 | HTRA1 |  |  | 3331 | -0.796 | -0.2129 | No |
| 87 | PMEPA1 |  |  | 3338 | -0.804 | -0.2024 | No |
| 88 | SPARC |  |  | 3356 | -0.822 | -0.1948 | No |
| 89 | COL3A1 |  |  | 3430 | -0.960 | -0.2009 | No |
| 90 | DPYSL3 |  |  | 3521 | -1.188 | -0.2083 | No |
| 91 | FBLN1 |  |  | 3539 | -1.262 | -0.1940 | No |
| 92 | SPP1 |  |  | 3541 | -1.286 | -0.1748 | No |
| 93 | CDH2 |  |  | 3543 | -1.297 | -0.1554 | No |
| 94 | CADM1 |  |  | 3581 | -1.591 | -0.1417 | No |
| 95 | TNC |  |  | 3586 | -1.618 | -0.1184 | No |
| 96 | IGFBP3 |  |  | 3588 | -1.623 | -0.0941 | No |
| 97 | COL1A2 |  |  | 3602 | -1.803 | -0.0704 | No |
| 98 | MGP |  |  | 3612 | -1.988 | -0.0429 | No |
| 99 | IGFBP2 |  |  | 3634 | -3.295 | 0.0011 | No |
Table: GSEA details [plain text format]

  

Fig 2: HALLMARK\_EPITHELIAL\_MESENCHYMAL\_TRANSITION: Random ES distribution      
 Gene set null distribution of ES for **HALLMARK\_EPITHELIAL\_MESENCHYMAL\_TRANSITION**
